# Supplementary figures and images for: Isolating and cryopreserving pig skin cells for single-cell RNA sequencing study
Source: PLoS One. 2022 Feb 17;17(2):e0263869. doi: 10.1371/journal.pone.0263869 (PMC8853494; doi:10.1371/journal.pone.0263869)

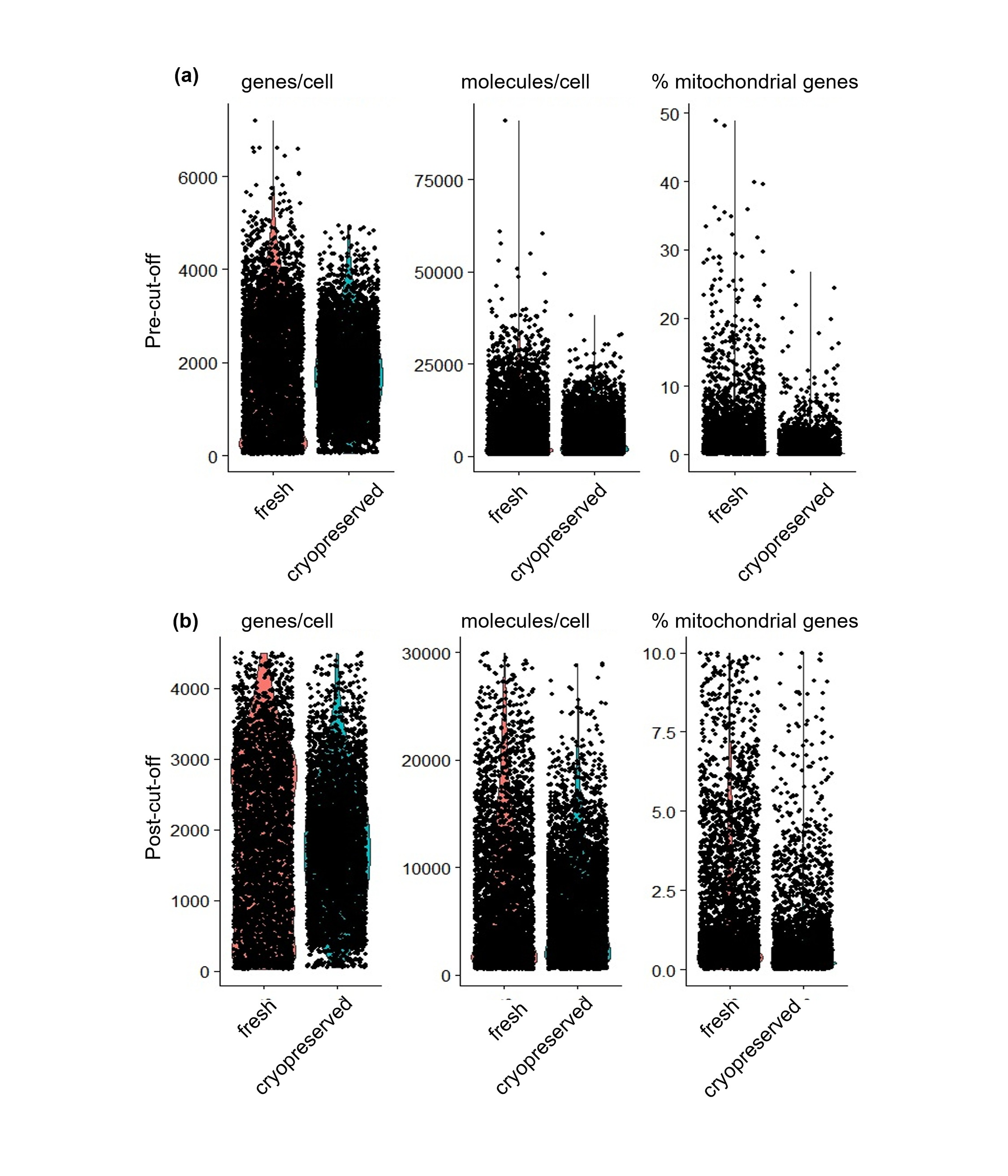

Supplement: S1 Fig — (a) pre-cut-off and (b) post-cut-off data are shown. 4500 genes/cell, 30,000 molecules/cell, and 10% mitochondrial genes are set as the maximum threshold to exclude the doublets, multiples, and low-quality single cells. Each dot represents one cell. (TIF) [file pone.0263869.s001.tif]

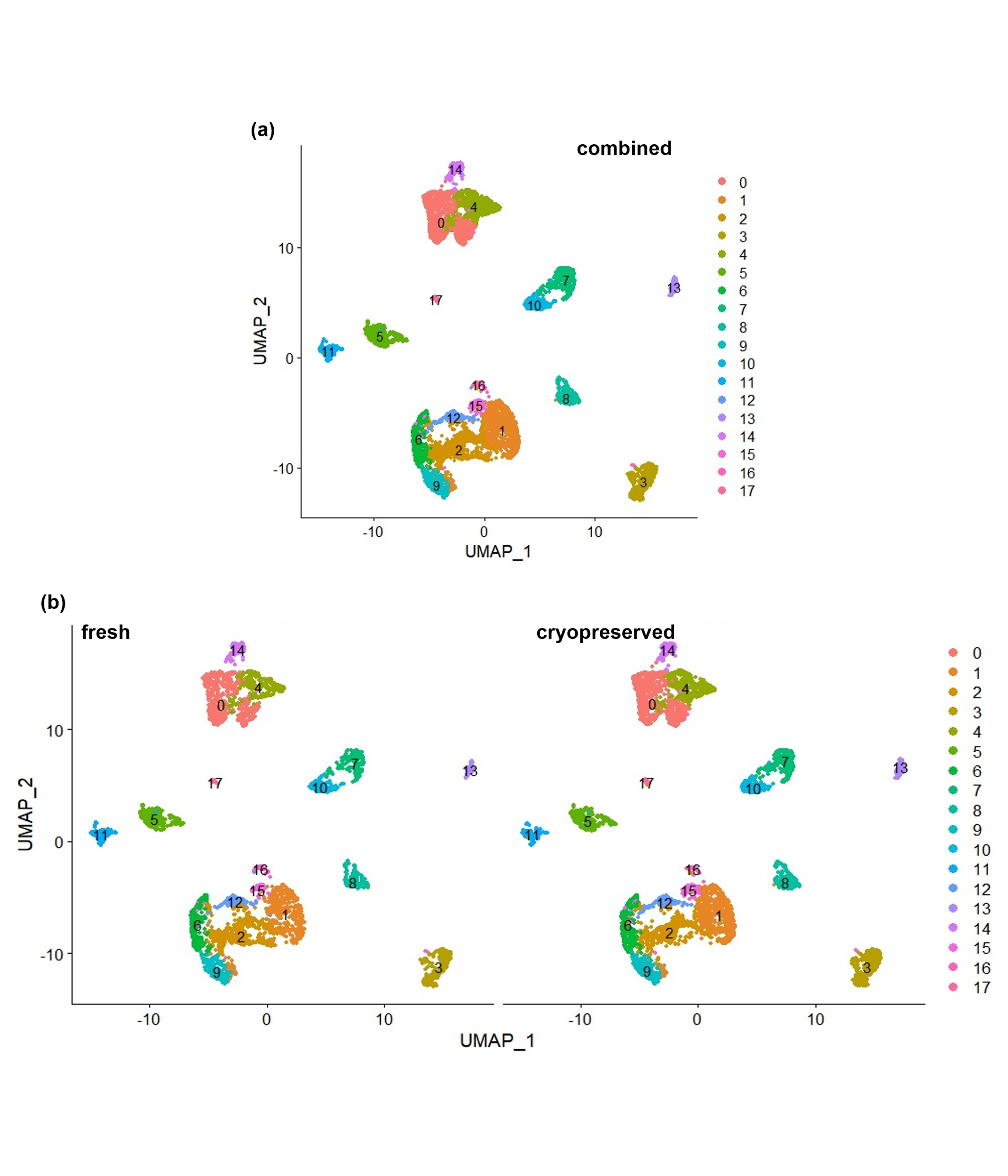

Supplement: S2 Fig — Clustering analysis of combined (a) and separated (b) fresh and cryopreserved samples shows that the two samples have similar clusters. (TIF) [file pone.0263869.s002.tif]

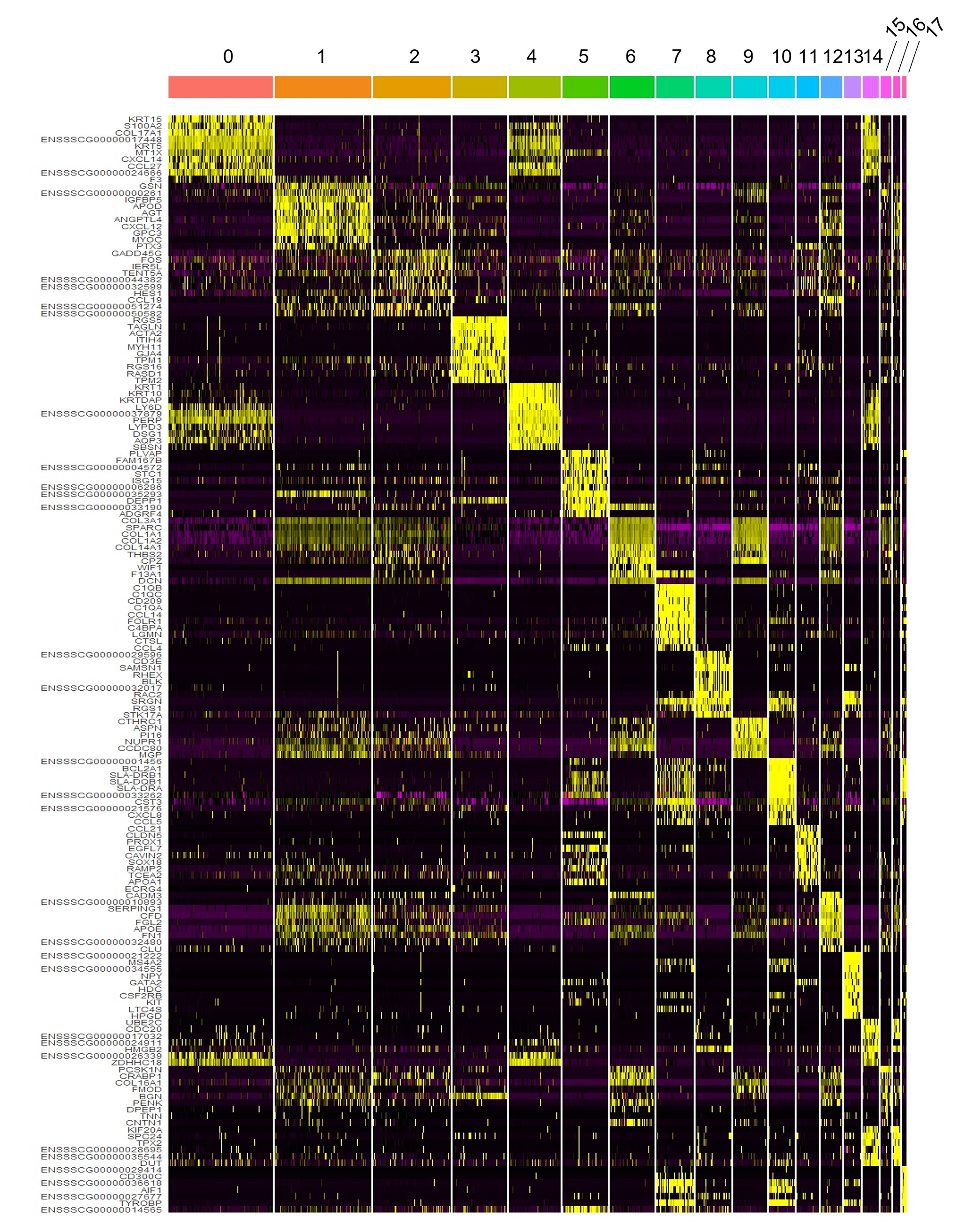

Supplement: S3 Fig — (TIF) [file pone.0263869.s003.tif]

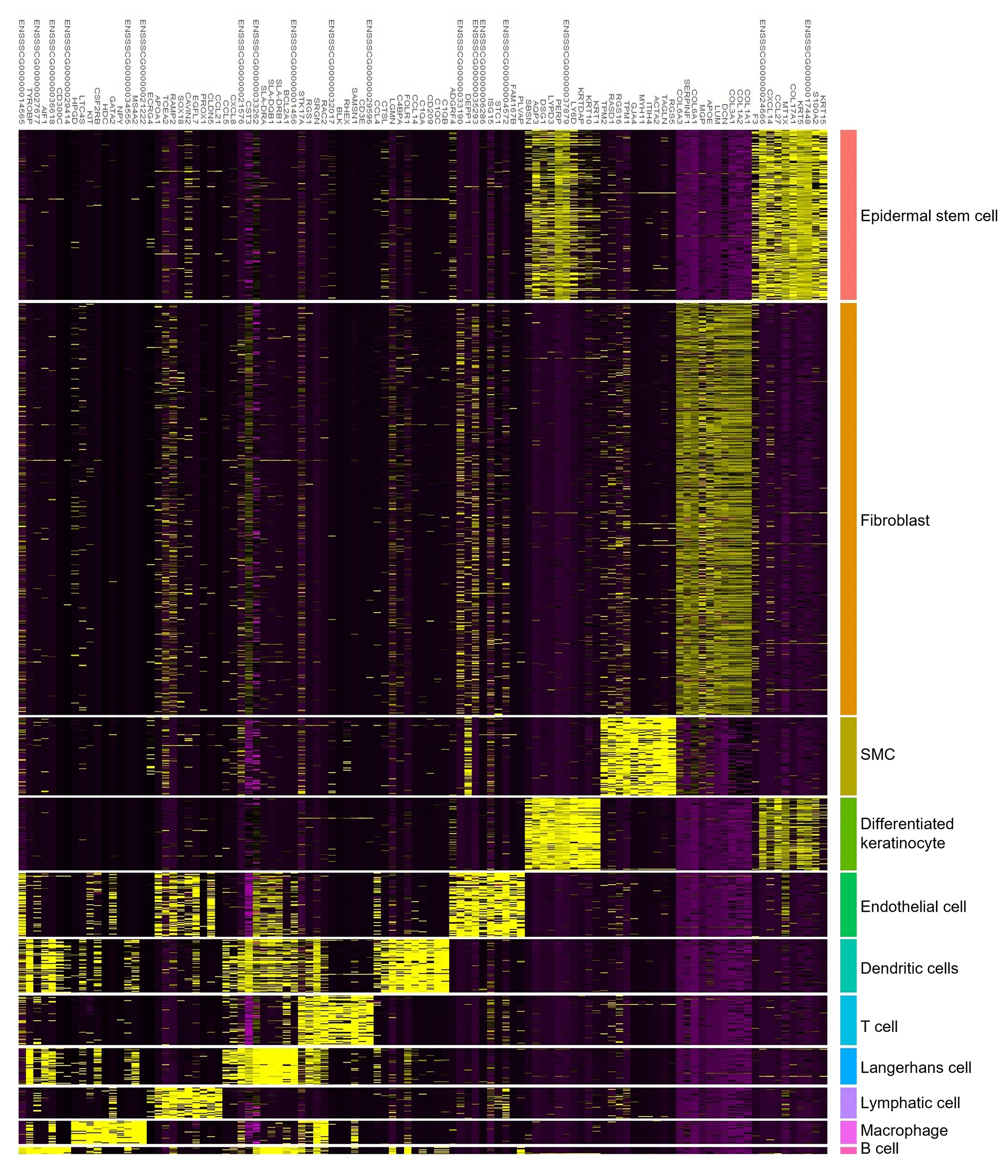

Supplement: S4 Fig — (TIF) [file pone.0263869.s004.tif]

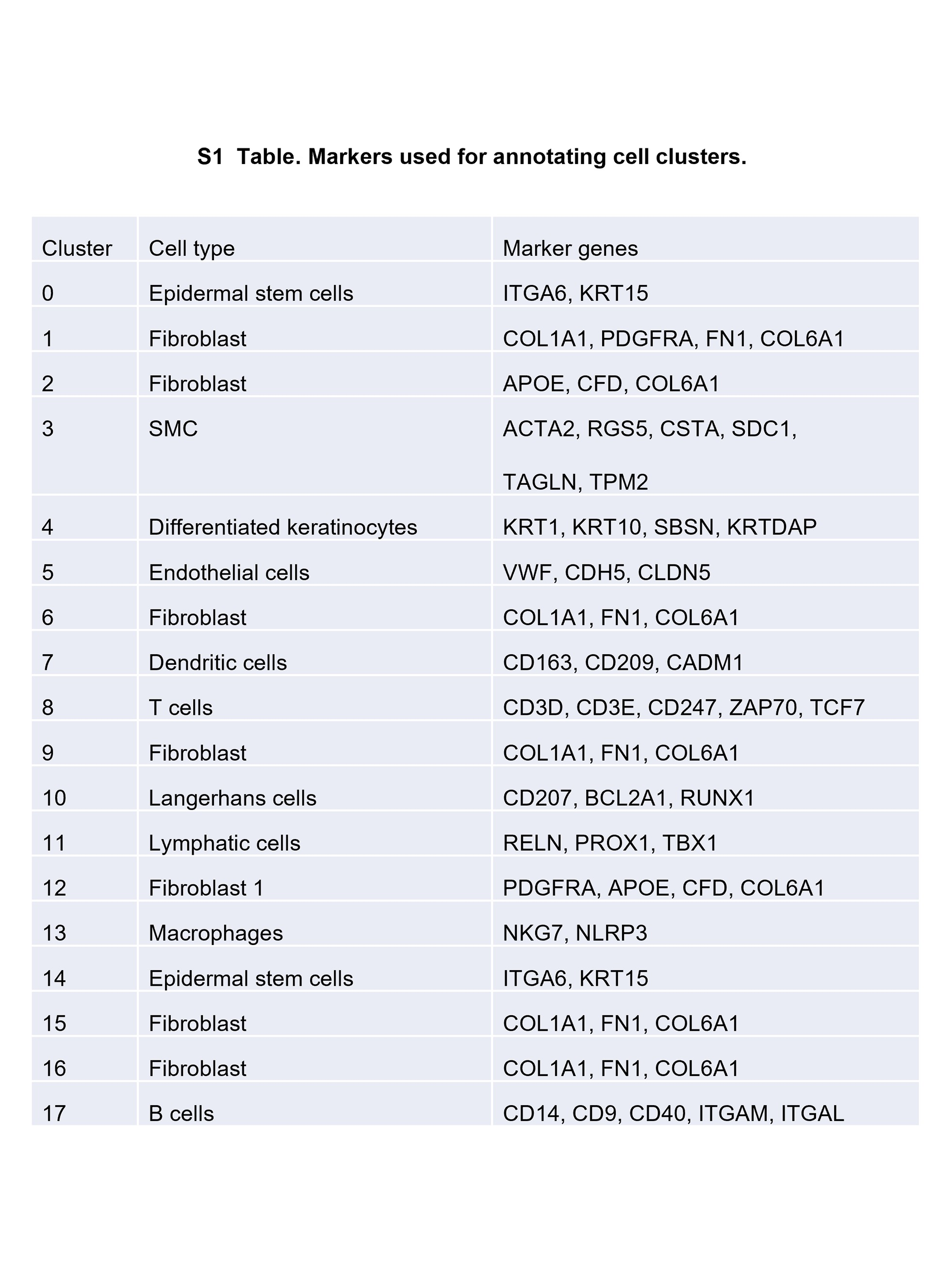

Supplement: S1 Table — (TIF) [file pone.0263869.s005.tif]
