# Supplementary material for: Isolating and cryopreserving pig skin cells for single-cell RNA sequencing study
Source: PLoS One. 2022 Feb 17;17(2):e0263869. doi: 10.1371/journal.pone.0263869 (PMC8853494; doi:10.1371/journal.pone.0263869)
Supplement: Attachment — Submitted filename: Response to Reviewers.docx [file pone.0263869.s006.docx]

**Response to reviewers (point by point responses to reviewers’ comments)**

**I: Major changes**

| Figure in the original manuscript | Corresponding Figure in the revised manuscript |
| --- | --- |
| Fig 4 | S2 Fig |
| Fig 5 | Fig 4 |
|  | Add new Fig 5 |
| Table 1 | S1 Table |
| Table 2 | Table 1 |
|  | Format the manuscript to comply with the PLOS ONE journal requirements |
|  |  |

**II: Point-to-Point rebuttal to editors’ and reviewers’ comments**

We first want to thank the editor and reviewers for the positive comments and thoughtful feedback, which have prompted many improvements. The followings are our Point-to-Point answers to the questions.

**Journal Requirements:**

1.  Please ensure that your manuscript meets PLOS ONE's style requirements, including those for file name.

**Response:** The manuscript has been formatted to comply with the PLOS ONE style requirements.

2. We note that you are reporting an analysis of a microarray, next-generation sequencing, or deep sequencing data set. PLOS requires that authors comply with field-specific standards for preparation, recording, and deposition of data in repositories appropriate to their field. Please upload these data to a stable, public repository (such as ArrayExpress, Gene Expression Omnibus (GEO), DNA Data Bank of Japan (DDBJ), NCBI GenBank, NCBI Sequence Read Archive, or EMBL Nucleotide Sequence Database (ENA)). In your revised cover letter, please provide the relevant accession numbers that may be used to access these data.

**Response:** Revised as suggested.

3.  We note that the grant information you provided in the ‘Funding Information’ and ‘Financial Disclosure’ sections do not match.

When you resubmit, please ensure that you provide the correct grant numbers for the awards you received for your study in the ‘Funding Information’ section.

**Response:** Revised as suggested.

4. PLOS requires an ORCID iD for the corresponding author in Editorial Manager on papers submitted after December 6th, 2016. Please ensure that you have an ORCID iD and that it is validated in Editorial Manager. To do this, go to ‘Update my Information’ (in the upper left-hand corner of the main menu), and click on the Fetch/Validate link next to the ORCID field. This will take you to the ORCID site and allow you to create a new iD or authenticate a pre-existing iD in Editorial Manager. Please see the following video for instructions on linking an ORCID iD to your Editorial Manager account: [https://www.youtube.com/watch?v=_xcclfuvtxQ](https://nam10.safelinks.protection.outlook.com/?url=https%3A%2F%2Fwww.youtube.com%2Fwatch%3Fv%3D_xcclfuvtxQ&data=04%7C01%7Cyxl6034%40psu.edu%7C6db5b7f709dd46d6409108d963755fec%7C7cf48d453ddb4389a9c1c115526eb52e%7C0%7C0%7C637650179432255456%7CUnknown%7CTWFpbGZsb3d8eyJWIjoiMC4wLjAwMDAiLCJQIjoiV2luMzIiLCJBTiI6Ik1haWwiLCJXVCI6Mn0%3D%7C1000&sdata=u4nsuCQ3lImUUJ0wBShQ8dOFEYwABRXt8pFABDyBNko%3D&reserved=0)

**Response:** Revised.

**Reviewer #1:** 

1) There are some typos in Figure 1B that should be corrected (Cry-thaw, FACs, MACs)

**Response:** Revised.

2) Figure 4 and Figure 5 are very duplicative. The figures could be combined, or figure 4 moved to supplemental data (or deleted).

**Response**: We moved Fig 4 to supplemental data (S2 Fig).

3) Figure 3a: the axis of the plot need to be labeled more clearly. Assuming that the data shown is the relative gene expression. Log transforming the data on such a plot would be a more conventional way to display the data, and highlighting some important genes would help with orientation.

**Response**: Revised following the suggestion.

4) The description of the trajectory analysis could use some refinement. As currently written the data is presented as demonstrating that cell differentiation occurs in both fresh and frozen cells. A more correct/helpful description of the data would be something along the lines of "the data quality obtained from both fresh and frozen cells was of sufficient quality to allow transcriptional trajectory/pseudotime analysis"

**Response**: Revised as suggested.

5) Population identification/annotation is a very important step in scRNAseq analysis, especially for novel tissue types and "non-standard" model systems. It might be helpful to move some of the linage-defining gene expression data from supplemental data to a primary figure, and perhaps use a dot plot to highlight key genes from each population.

**Response**: Following the suggestion, we added Fig 5.

**Reviewer #2:**

1# If I want to summarize the content of this manuscript, there would be three key points – i) Isolation of single cell from Pig skin, ii) Cryo-preservation of that single cell and iii) RNA sequencing of that single pig skin cell. scRNA-seq is a common and established method of gene expression analysis if the single cell is available. Thus, the main part of this manuscript is the isolation and preservation of single pig skin cells. Unfortunately, the authors highlighted scRNA-seq in the abstract not the isolation methodology. In the Introduction section the authors did the same approach. They need to rewrite the Abstract and Introduction in a more presentable format. Additionally, the last paragraph of introduction needs to be removed. Introduction should not contain any methodology or results. Instate of that the authors need to present their aim of this research.

**Response:** Thanks for the comments. We revised the manuscript as suggested.

2# Which one is correct – pigskin or pig skin? Authors need to follow only one style throughout the manuscript.

**Response**: Pig skin is used in the revised manuscript.

3# In the Materials and Methods, the authors claimed only Methods. Does it compatible with Plos One? The first sub-heading in this section is entitled as ‘Pig skins’. Does it mean anything? It could be ‘Harvesting of pig skin’. Writing of a manuscript and writing of lab note is different. Authors are suggested to recheck all the sub-headings in this manuscript and change them in the more presentable format, if required. Additionally, they are suggested to follow the international format of writing the company names for any chemicals and instruments throughout the manuscript.

**Response:** Revised as suggested.

4# The isolation of single cell from pig skin is the main part of this manuscript. So, authors need to describe in detail procedure in the Materials and Methods. The present description is not satisfactory.

**Response:** We added details of the isolation process.

5# Author wrote ‘Libraries were sequenced using Illumina technology.’ Does they have sequencer in their Lab? If so, then provide the company name. If not, then mention the commercial service name.

**Response**: Revised as suggested.

6# Authors need to recheck the suitability of data deposition statement in the body text. If nor, they are requested to remove the ‘Data and code availability’ section from the text.

**Response:** Removed from the text.

7# Which one is correct – liquid N2 or liquid N2? Authors need to follow only one style throughout the manuscript. Furthermore, they need to check for cryopreservation or cryo-preservation. And so on ……….

**Response:** Changed all the “liquid N2” to “liquid N_2_”. In addition, we replaced “frozen” by “cryopreserved”.

8# It is well established that 10% DMSO provides good preservation of live cells. The authors of this manuscript also used the same procedure. However, they want to claim that they did something new. They are requested to show every point they have modified in the general procedure. Additionally, several times they used the term ‘DMSO-based cryopreservation’. For example, they claim that DMSO-based cryopreservation preserved gene expression. What does it mean? Does it mean that among several cryopreservation techniques DMSO-based cryopreservation showed optimum result? Does it mean that DMSO-based cryopreservation is mandatory for pig skin cell? The authors need to show a comparative analysis among different preservation techniques if they want to claim so. Additionally, they need to provide a positive control (either human skin/rodent skin cells) to compare.

**Response:** Thanks for the comments.

First, we changed the “DMSO-based cryopreservation” to “cryopreservation in 90% FBS +10% DMSO” to avoid the confusion.

Second, we did not claim: 1) we invented a new cryopreservation method, or improved/modified the DMSO-based cryopreservation method; 2) the DMSO-based cryopreservation is better than other cell preservation methods.

**The goal of this work** is to find, demonstrate, validate a robust method/protocol for isolating and preserving single pig skin cells for scRNA-Seq study. **To our best knowledge, there have been no reports** on how to prepare and preserve single cells from pig skin tissue for scRNA-Seq in the literature. We showed that using our reported method/protocol, high-quality scRNA-Seq data could be generated to identify the major skin cell types. We believe this report is very valuable for the skin research scientific community.

9# In summary, the authors isolated single cells from pig skin, cryopreserved and compare it with fresh cell in genomic level. Thus, the merit goes to the impact of preservation in genomic level. So far, this manuscript is pioneer to isolating single cells from pig skin. In this regards it has novelty. But if they want to show the impact of preservation then the present format of manuscript is not suitable. They need to add positive control and comparison data for different preservation techniques. And rewrite the manuscript in particular manner.

**Response:** same as 8#.

10# Rigorous English editing is required.

**Response:** Revised as the suggestion.
