# Supplementary material for: Isolating and cryopreserving pig skin cells for single-cell RNA sequencing study
Source: PLoS One. 2022 Feb 17;17(2):e0263869. doi: 10.1371/journal.pone.0263869 (PMC8853494; doi:10.1371/journal.pone.0263869)
Supplement: Attachment — Submitted filename: Response to Reviewers(2).docx [file pone.0263869.s007.docx]

**Response to reviewers (point by point responses to reviewers’ comments)**

**I：Point-to-Point rebuttal to editors’ and reviewers’ comments**

Again we want to thank the editor and reviewers for the comments and feedback. The followings are our Point-to-Point answers (highlighted in Blue) to the questions.

**Journal Requirements:**

Please review your reference list to ensure that it is complete and correct. If you have cited papers that have been retracted, please include the rationale for doing so in the manuscript text, or remove these references and replace them with relevant current references. Any changes to the reference list should be mentioned in the rebuttal letter that accompanies your revised manuscript. If you need to cite a retracted article, indicate the article’s retracted status in the References list and also include a citation and full reference for the retraction notice.

All have been done.

**Reviewers' comments:**

Reviewer #2: A) In response to my query #8 the authors answered as follows –

1) We invented a new cryopreservation method, or improved/modified the DMSO-based cryopreservation method;

*Observation: The authors used three keywords invention, improvement and modification. Which one is appropriate? Do they really invent a new protocol??? I think they just modified the existing protocol for pig skin cell isolation. If so, then they need to let us know in which points they have changed. Furthermore, does the modification bring better result? If so, then they need to show us both results in a comparison manner. We need to understand that their modification is meaningful.*

2) The DMSO-based cryopreservation is better than other cell preservation methods.

*Observation: We all agree with this statement. Everybody knows that the DMSO-based cryopreservation method is the best one. Then, what is new here the author is claiming. Why the authors are highlighting DMSO-based cryopreservation for pig cells? This would be a normal procedure for cell preservation.*

**Response**: There is some miscommunication here. We strongly agree with the reviewer about the observations. In our response to to query #8, we said:

*“Second,* ***we did not claim:*** *1) we invented a new cryopreservation method, or improved/modified the DMSO-based cryopreservation method; 2) the DMSO-based cryopreservation is better than other cell preservation methods”*

We said “**we did not claim**”.

B) The reviewer is not satisfied with the answer for #9. The authors need to organize their manuscript in a manner that they just isolated single cells from pig skin.

**Response**: Sorry that we did not have a clear answer in our last response. We did re-ogranize the manuscript in a manner suggested by the reviewer in the previous revision.

Again, thanks for all the suggestions!
